# Incarcerated Patients Presenting to an Academic Emergency Department: A Retrospective Review Using Payor Source for Identification

**DOI:** 10.7759/cureus.113579

**Published:** 2026-07-29

**Authors:** Alexander Fenn, Maglin Halsey-Nichols, Matthew Scholer, Anna E Waller, Pasangi Perera, Justin G Myers

**Affiliations:** 1 Department of Emergency Medicine, University of North Carolina at Chapel Hill, Chapel Hill, USA; 2 Department of Epidemiology, Gillings School of Global Public Health, University of North Carolina at Chapel Hill, Chapel Hill, USA

**Keywords:** access to healthcare and health outcomes of vulnerable populations, correctional health, electronic health records, emergency medicine, prisoners

## Abstract

Background: Emergency departments (EDs) serve an important role in the care of patients who are imprisoned (incarcerated) in the federal and state prison systems, and the University of North Carolina Healthcare (UNCH) System helps provide care to these patients. Our aim was to identify and characterize this marginalized patient population by payer source, compare it with the general ED population, and, ultimately, seek ways to improve ED and follow-up care.

Methods: This was a hypothesis-generating study utilizing two separate ED sites: a tertiary referral academic ED and a smaller, academically affiliated community hospital. Retrospective data were sourced from the Epic Electronic Health Record via the Carolina Data Warehouse for Health, part of the North Carolina Translational and Clinical Sciences Institute. All ED encounters involving incarcerated individuals were obtained from January 1, 2018, through December 31, 2024. Inmate encounters were identified via a distinct payer source, the NC Department of Public Safety. Descriptive statistics, including means and percentages, were generated using Python (Python Software Foundation, Beaverton, Oregon) and pandas (The pandas development team, NumFOCUS, Austin, Texas).

Results: A total of 555 encounters involving 478 unique patients were identified. The majority of patients presenting from NC prison facilities were male (92.4% of encounters), compared with 43.7% of ED patients overall. The average age was similar (46.4 versus 49.7 years), but a smaller proportion of patients from prison were younger (18-24 years old) or older (over 65 years old). A higher percentage of patients presenting from prison were identified as Black or African American (44.9% versus 29.6%). The most common chief complaints were abdominal pain, chest pain, and “abnormal lab,” compared with abdominal pain, chest pain, and shortness of breath in the general ED population.

Conclusion: Challenges exist in retrospectively identifying incarcerated patients in the ED for research. Using a distinct payer source, we identified and characterized a cohort of incarcerated patients and compared them with other ED patients. Future work will investigate how to improve ED and follow-up care for this unique patient population.

## Introduction

In the United States, nearly two million people are incarcerated in prisons or jails, representing the largest incarcerated population of any country in the world. This figure includes approximately 1.2 million individuals held in state and federal prisons and over 600,000 in local jails at any given time [1]. The United States incarcerates people at a rate of roughly 580 per 100,000 residents, a rate that far exceeds that of other high-income nations and has prompted widespread concern about the public health consequences of mass incarceration [2,3]. 

Incarceration is not experienced equally across the population. It disproportionately affects individuals from racially minoritized groups, particularly Black/African Americans, as well as those who are socioeconomically disadvantaged [3]. Black/African American populations are incarcerated at approximately five times the rate of White American populations. Individuals with lower levels of educational attainment and income are substantially overrepresented in the incarcerated population as well. These disparities in exposure to the criminal legal system are deeply intertwined with and exacerbate existing disparities in health outcomes [4]. 

People who are incarcerated have elevated rates of numerous health conditions compared to the general population. Infectious diseases such as hepatitis B, hepatitis C, and HIV are significantly more prevalent in correctional settings, as are mental health conditions and substance use disorders [5,6]. Chronic conditions, including diabetes, cardiovascular disease, stroke, and chronic lung disease, also occur at higher rates among people with current or prior incarceration [7]. Furthermore, mortality rates among incarcerated individuals exceed those of the general population for homicide, suicide, and cancer [8]. 

Importance 

Emergency departments (EDs) play an important role in the acute care of people who are incarcerated. When health care needs exceed the capacity of on-site correctional medical services, incarcerated individuals are transported to community EDs for evaluation and treatment. In this context, EDs often function as the primary access point for specialized diagnostic and therapeutic services that are unavailable within correctional facilities [9,10] 

Goals of this investigation 

The ability to accurately identify incarcerated individuals in electronic health record (EHR) data is essential for understanding this population’s health care utilization patterns and improving care delivery. However, this identification has historically been challenging. ICD-10-CM (International Classification of Diseases, Tenth Revision, Clinical Modification) diagnostic codes related to incarceration have been shown to have very low sensitivity for detecting incarceration status. Recent efforts have explored natural language processing (NLP) techniques to identify incarceration-related terms in clinical notes, with promising but resource-intensive results [11]. To our knowledge, no prior research has examined the use of payor source to identify incarcerated patients in ED encounter data. Because the costs of health care for state prisoners are typically borne by the state department of corrections and billed through identifiable payor codes, payor source may represent a practical, low-cost, and potentially replicable method for identifying this population in administrative and clinical data. We anticipated that payor source would perform well relative to note-based approaches because correctional billing is recorded as structured EHR data, though we recognized at the outset that this approach would identify only individuals whose care was billed through a correctional payer and would not, by itself, confirm incarceration status. 

North Carolina has a prison incarceration rate slightly below the national average [12]. The University of North Carolina Health Care (UNC Health) system consists of 16 hospitals across 20 campuses throughout North Carolina and provides care to individuals incarcerated in nearby state correctional facilities. The aim of this study was to use payor-source data to identify and characterize ED encounters among incarcerated people at two EDs within the UNC Health system, compare this cohort with the general adult ED population served by the same EDs, and identify opportunities to improve emergency and follow-up care for this medically underserved population. 

## Materials and methods

Study design and IRB approval 

This was a retrospective, observational cohort study. The study was reviewed and approved by the Institutional Review Board at the University of North Carolina at Chapel Hill. 

Setting 

Data for this study were sourced from the Epic EHR via the Carolina Data Warehouse for Health (CDWH), part of the North Carolina Translational and Clinical Sciences (NC TraCS) Institute. The CDWH is a clinical data repository that aggregates de-identified patient data from the UNC Health system for research purposes. The study included encounter data from two EDs within the UNC Health system: (1) a quaternary care, Level 1 trauma center and academic ED located in a suburban setting, which evaluates more than 65,000 patients per year, and (2) a smaller, academic-affiliated community hospital ED located in a rural/suburban setting, which evaluates more than 25,000 patients per year. Together, these two EDs serve a diverse patient population across central North Carolina and receive patients transported from multiple state correctional facilities in the region. 

Cohort selection 

All ED encounters involving incarcerated individuals from January 1, 2018, through December 31, 2024, were identified, spanning seven complete calendar years. Encounters involving incarcerated individuals were identified through distinct payer sources in the EHR billing data. Specifically, encounters billed to the North Carolina Department of Public Safety or associated correctional payer codes were flagged for inclusion. This approach relies on the Department of Public Safety’s financial responsibility for the health care of individuals incarcerated in state facilities, which creates an identifiable billing pattern within the EHR. The payer sources used to identify the incarcerated cohort were “NC Department of Public Safety,” “Department of Public Safety,” and “Federal Correctional.” These categories were selected based on prior studies of incarcerated cohorts within the Carolina Data Warehouse for Health. Because this approach reflects the current institutional standard for identifying incarcerated individuals, it was not formally validated against an external reference standard.

**Figure 1:**
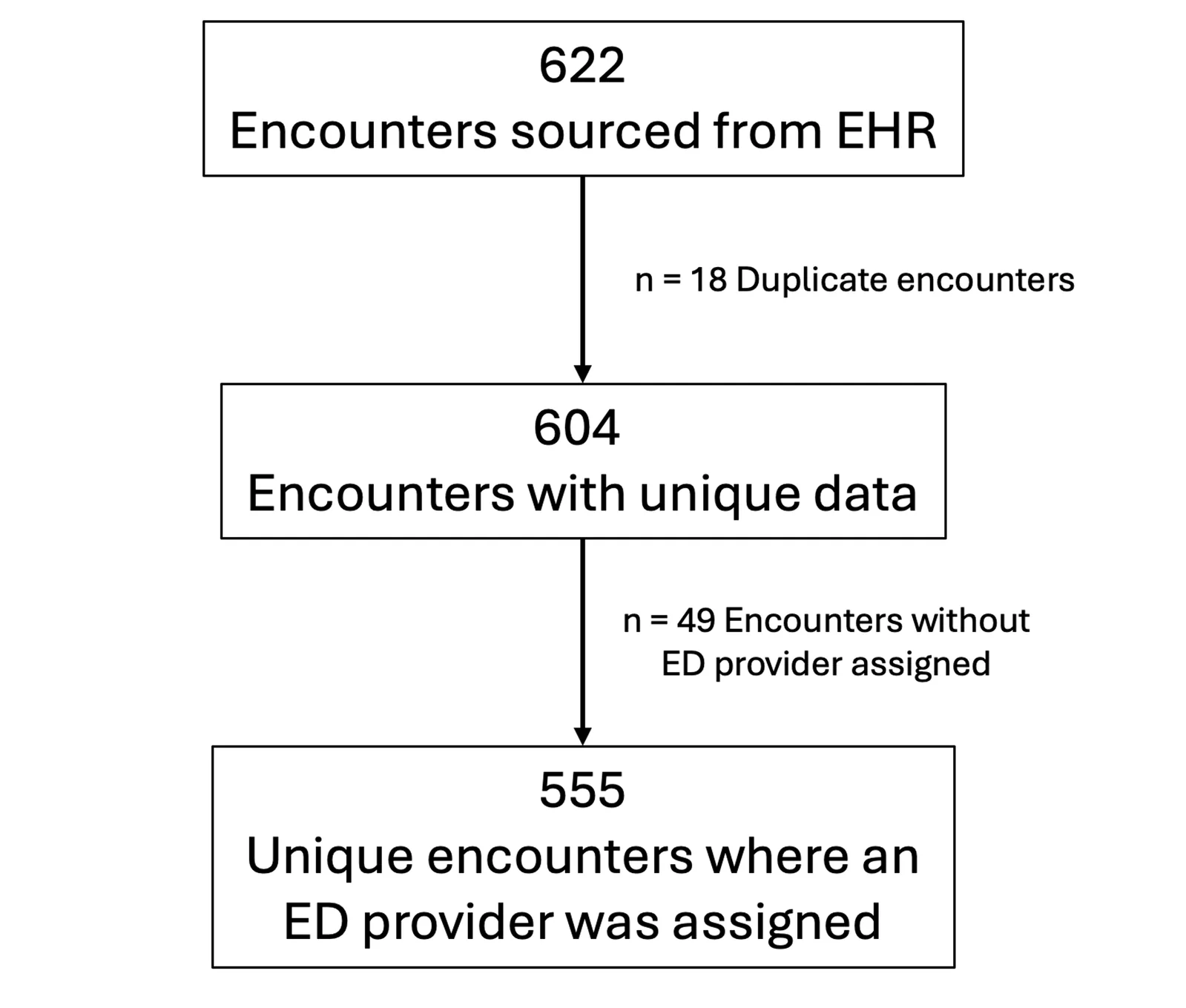
Patient inclusion flowchart EHR: electronic health record, ED: emergency department.

A total of 622 encounters involving incarcerated individuals were initially identified using this method. After detailed review and data cleaning, 18 duplicate encounter records (i.e., instances in which the same encounter was recorded twice) were removed. An additional 49 encounters were excluded because no ED provider was assigned. A substantial proportion of these excluded encounters involved patients presenting for legal blood draws conducted under police custody. Such cases require an ED encounter to be created in the EHR for documentation and billing purposes but do not follow the typical ED workflow involving evaluation by a clinician. Because these encounters did not constitute traditional clinical ED visits, they were excluded from the analytic cohort and were not examined further. Figure 1 illustrates the analytic cohort derivation process. The final analytic cohort comprised 555 encounters involving 478 unique patients. 

Data analysis

Statistical analyses of the incarcerated cohort were performed using Python (version 3.13, Python Software Foundation, Beaverton, Oregon) and the pandas package (The pandas development team, NumFOCUS, Austin, Texas). Descriptive statistics were calculated for all variables of interest, including demographic characteristics (age, sex, and race/ethnicity), Emergency Severity Index (ESI) triage level, patient disposition (admission, discharge, transfer, or leaving against medical advice), ED length of stay (LOS), chief complaints, past medical history, and 30-day return visit rates. Continuous variables are reported as means with standard deviations and medians, whereas categorical variables are reported as frequencies and percentages.

For comparative analysis, aggregate cohort statistics for all adult (aged ≥18 years) ED encounters at the same two EDs during the identical period (January 1, 2018, through December 31, 2024) were obtained using the Epic SlicerDicer analytical tool. SlicerDicer is a self-service data exploration tool within the Epic electronic health record system that allows users to query and aggregate clinical data across patient populations. Because SlicerDicer provided only aggregate-level comparator data, formal statistical testing and adjusted analyses were not performed. All comparisons between the two cohorts are descriptive.

## Results

A total of 555 ED encounters involving 478 unique incarcerated patients were identified over the seven-year study period. Table 1 presents the demographic characteristics of ED encounters involving incarcerated individuals and all adult ED encounters during the study period. The incarcerated cohort was predominantly male, with 513 encounters (92.4%) involving male patients, compared with 228,227 (43.7%) of the 521,877 adult ED encounters. Female patients accounted for 42 encounters (7.6%) in the incarcerated cohort, compared with 293,589 (56.3%) in the general adult ED population. The mean patient age was similar between the incarcerated cohort (46.44 years, SD = 13.83) and the general adult ED population (49.66 years, SD = 19.81). However, the age distributions differed: smaller proportions of encounters involving incarcerated individuals were in the youngest age group (18-24 years: 3.6% vs. 11.5%) and the oldest age groups (65-74 years: 6.5% vs. 12.5%; ≥75 years: 2.7% vs. 12.8%), whereas a higher proportion were in the middle age range of 25-64 years. The largest age category in the incarcerated cohort was 45-54 years (22.9%), whereas that in the general population was 25-34 years (16.6%). For context, the North Carolina prison population numbered 31,932 at the end of the state fiscal year 2023-2024, and the largest age category among incarcerated males was 30-39 years; within that group, 52% were Black/African American and 39% were White. The concentration of our cohort in the middle adult age ranges and the higher proportion of Black/African American patients are broadly consistent with this state-level distribution. 

**Table 1:** Cohort demographics

Sample Characteristic	Inmate Encounters (N = 555)	All Adult ED Encounters (N = 521,877)
Demographics		
Male	513 (92.4%)	228,227 (43.7%)
Female	42 (7.6%)	293,589 (56.3%)
Unknown	N/A	61(<0.1%)
Age in years, mean (SD)	46.44 (13.83)	49.66 (19.81)
18-24	20 (3.6%)	60,042 (11.5%)
25-34	119 (21.4%)	86,447 (16.6%)
35-44	125 (22.5%)	80,057 (15.5%)
45-54	127 (22.9%)	81,070 (15.5%)
55-64	113 (20.4%)	81,474 (15.6%)
65-74	36 (6.5%)	65,129 (12.5%)
75+	15 (2.7%)	66,858 (12.8%)
Race/ethnicity		
White	255 (45.9%)	292,348 (56%)
Black	249 (44.9%)	154,565 (29.6%)
Other race	24 (4.3%)	60,855 (11.7%)
Two or more races	13 (2.3%)	N/A
American Indian/Alaskan Native	5 (.9%)	5,125 (1%)
Asian	1 (.1%)	4,712 (0.9%)
Unknown/Prefer not to answer/None of the above	8 (1.4%)	11,127 (2.1%)

Regarding race and ethnicity, a higher proportion of patients in the incarcerated cohort identified as Black/African American (44.9% vs. 29.6%) than in the general adult ED population. A lower proportion of incarcerated patients identified as White (45.9% vs. 56.0%). American Indian/Alaska Native patients accounted for 0.9% of incarcerated encounters and 1.0% of all adult ED encounters, whereas Asian patients accounted for 0.1% and 0.9%, respectively.

Table 2 presents patient disposition data for ED encounters. A slightly higher proportion of incarcerated patients were discharged from the ED (73.3%) than in the general adult ED population (65.2%), whereas a lower proportion were admitted to the hospital (22.2% vs. 27.1%). The proportion of patients leaving against medical advice (AMA) was identical in both groups (0.9%). 

**Table 2:** Cohort dispositions *includes other dispositions such as LWBS before triage, sent to L+D, expired.

	Inmate	Adult ED
Admit	123 (22.2%)	141,558 (27.1%)
Discharge	407 (73.3%)	340,331 (65.2%)
Left without being seen (LWBS) after triage	N/A	18,806 (3.6%)
Against medical advice (AMA)	5 (0.9%)	4,557 (0.9%)
Transfer to another hospital/facility/ED	20 (3.6%)	4,148 (0.8%)
Other/None of the above*	N/A	12,477 (2.4%)

Table 3 presents ESI triage-level data. The incarcerated cohort had a higher proportion of encounters triaged at ESI level 3 (moderate acuity), with 383 encounters (69.0%), compared with 306,465 (58.7%) of all adult ED encounters. Incarcerated patients were less frequently triaged at ESI level 2 (15.7% vs. 19.4%) and ESI level 4 (10.8% vs. 16.6%). The proportions of encounters triaged at ESI level 1 (highest acuity: 2.7% vs. 2.5%) and ESI level 5 (lowest acuity: 1.6% vs. 2.1%) were similar between the groups. 

**Table 3:** Cohort Emergency Severity Index (ESI) triage scores

ESI	Inmate	Adult ED
1	15 (2.7%)	13,289 (2.5%)
2	87 (15.7%)	101,002 (19.4%)
3	383 (69.0%)	306,465 (58.7%)
4	60 (10.8%)	86,565 (16.6%)
5	9 (1.6%)	11,190 (2.1%)
Unknown	1 (0.2%)	3,366 (0.6%)

Table 4 presents ED length-of-stay (LOS) data. The median ED LOS was slightly shorter for incarcerated patients than for the general adult ED population (4.70 hours vs. 5.33 hours). The mean LOS was also shorter for the incarcerated cohort (7.06 hours, SD = 8.82) than for the general adult ED population (8.55 hours, SD = 25.52). 

**Table 4:** Cohort length of stay (LOS)

Index	Inmate LOS (hours)	Adult ED LOS (hours)
Mean	7.06 (SD 8.82)	8.55 (SD 25.52)
Median	4.7	5.33

**Figure 2:**
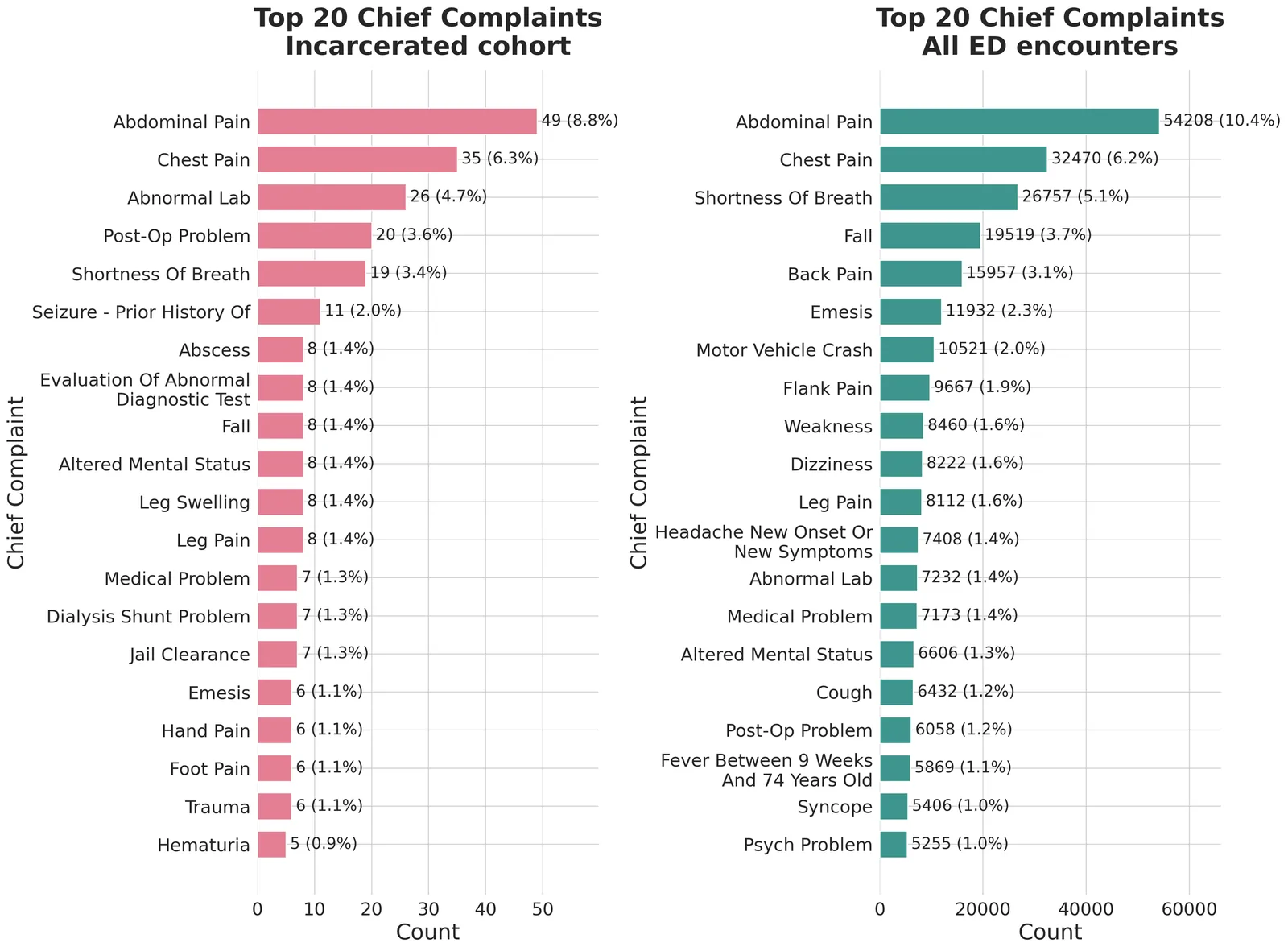
Top 20 chief complaints in the incarcerated cohort compared with the top 20 chief complaints out of all ED encounters, during the study period

Both incarcerated patients and patients in the general adult ED population most frequently presented with chief complaints of abdominal pain or chest pain. However, ED encounters involving incarcerated individuals included a chief complaint of “abnormal labs” at more than three times the rate observed in the overall ED population, suggesting a distinct pattern of referral from correctional facilities for follow-up of laboratory results, although the dataset did not capture the specific laboratory values or abnormalities associated with these encounters. With respect to past medical history, incarcerated patients had rates of hypertension, type 2 diabetes mellitus, and gastroesophageal reflux disease (GERD) similar to those observed in the general adult ED population. Notably, a relatively high proportion of incarcerated patients had a documented history of hepatitis C (5.65%), consistent with the known elevated prevalence of hepatitis C in correctional settings (Figure 2).

Approximately 1 in 20 ED encounters in the incarcerated cohort (4.5%) were return visits occurring within 30 days of a prior visit. Return visits were identified across the two study EDs (i.e., the patient returned to either of the two EDs in the study); visits to facilities outside these two sites could not be captured because of dataset constraints. Of these return visits, more than half occurred within 72 hours of the initial encounter. Review of the chief complaints associated with these return visits revealed that nearly all were the same as or clinically related to the chief complaint at the initial visit. Examples included a patient returning with chest pain after an initial visit for chest pain and a patient presenting with a wound infection after a prior visit for a chronic leg ulcer. This pattern may reflect incomplete resolution of the initial presenting problem or inadequate follow-up care between ED visits.

## Discussion

This study demonstrates the feasibility of using payor source data to identify a cohort of incarcerated patients within EHR-derived ED encounter data. To our knowledge, this is the first study to use payor source as the primary method for identifying incarcerated individuals in EHR-derived ED data. Even in one of the largest U.S. national datasets, the National Emergency Department Sample (NEDS), payor source is used to define ED cohorts; however, the available payor categories do not include the Department of Corrections. As a result, incarcerated patients are likely grouped under “Other,” limiting the utility of NEDS for identifying and studying this population [13]. Over the seven-year study period, we identified 555 ED encounters involving 478 unique incarcerated patients, a sufficient sample to characterize the demographic profile, acuity, disposition, and length of stay of this population. Given the unique medical and psychosocial needs of people who are incarcerated, the ability to reliably identify this population within clinical data systems is an important step toward improving care delivery and health outcomes for a medically underserved group. These findings support the feasibility of payor source identification rather than its diagnostic accuracy; further validation against a reference standard is needed before the method can be considered comprehensive for identifying incarcerated patients across health systems.

The payor source approach described here offers several advantages over previously described methods for identifying incarcerated individuals in clinical data. Natural language processing (NLP) models developed and validated to identify incarceration status from ED notes at a single academic center substantially outperformed ICD-10-CM codes, which showed very low sensitivity for detecting a history of incarceration [11]. Although NLP approaches show considerable promise, they require significant computational infrastructure, expertise in machine learning, and access to clinical notes, resources that may not be available at all institutions. Furthermore, payor source methods identify individuals who are currently incarcerated, whereas NLP approaches may detect both current and prior incarceration. Accordingly, these methods address different use cases rather than directly competing with one another. The payor source approach offers a straightforward, low-cost, and immediately replicable method that can be applied using standard administrative and billing data fields available in virtually all EHR systems. This approach does not require access to clinical notes, making it scalable across diverse health system settings. For example, Venkatesh et al. examined health care access and ED disposition patterns according to patients’ insurance status, finding that certain payor categories were associated with more vulnerable patient populations [14]. A bespoke, trained large language model was used to identify patients with a history of incarceration; however, it did not distinguish current incarceration status, limiting its applicability to the present study’s aims [15]. Future research should evaluate whether combining payor source identification with NLP-based approaches improves the accuracy of incarceration status ascertainment, potentially offering a complementary strategy that maximizes identification while minimizing resource requirements.

The demographic profile of our incarcerated cohort aligns with national data on incarcerated populations and findings from prior studies of ED utilization by people who are incarcerated. The predominance of male patients (92.4%) is consistent with the gender distribution within prisons nationally. The overrepresentation of Black/African American patients (44.9%) relative to the general ED population (29.6%) reflects well-documented racial disparities in incarceration rates in both North Carolina and the United States more broadly [4]. These findings underscore the intersection of mass incarceration and racial health inequities and reinforce the importance of culturally responsive, bias-aware care for this population in ED settings. 

Our finding that incarcerated patients were most frequently triaged at ESI level 3 (69.0%) suggests that most of these encounters represented moderate-acuity presentations requiring multiple resources for evaluation and management. The concentration at ESI level 3, with relatively lower proportions at ESI levels 4 and 5, may reflect institutional thresholds for transferring incarcerated individuals to external EDs: correctional facilities likely manage lower-acuity complaints through on-site health services and reserve ED transport, which involves considerable logistical coordination and security staffing, for conditions perceived as moderately or highly acute. Similar proportions of ESI level 1 encounters in the incarcerated and general populations suggest that the most critically ill patients are transferred to the ED regardless of incarceration status, as would be expected. Although these conclusions are reasonable, the paucity of national data on incarcerated ED populations and their presenting acuity levels limits opportunities for meaningful comparison.

The elevated rate of hepatitis C in the past medical history of incarcerated patients (5.65%) is consistent with the well-documented higher prevalence of hepatitis C in correctional settings. Because ED visits represent a key point of contact between incarcerated individuals and the broader health care system, these encounters may offer important opportunities for hepatitis C screening and linkage to care. Incarcerated patients may not always meet traditional targeted hepatitis C screening criteria, such as age-based risk, injection drug use, certain tattoos or piercings, or a history of higher-risk blood transfusion, as outlined in CDC and USPSTF guidance [16,17]. This limitation underscores recent evidence demonstrating that non-targeted hepatitis C screening in urban EDs is more effective at identifying new HCV cases than targeted approaches [18]. Moreover, both the CDC and USPSTF now recommend universal HCV screening for adults, positioning the ED as a viable setting for broad-based screening implementation.

Similarly, the finding that “abnormal labs” was a chief complaint at more than three times the rate observed in the general ED population raises questions about the nature of these encounters. Additional investigation is needed to determine the clinical circumstances underlying ED encounters categorized as “abnormal labs” and whether alternative models of evaluation, such as enhanced on-site correctional health services or telemedicine, may be appropriate in some settings.

To our knowledge, return ED visits among incarcerated patients have not been well described. Our 30-day return visit rate of 4.5%, with the majority occurring within 72 hours and involving a related chief complaint, suggests potential gaps in follow-up care after ED discharge. This rate is notable in the context of correctional health care, where barriers to continuity of care between the discharging ED and the receiving correctional facility’s health services are well documented. Although reasons for return visits were not analyzed beyond chief complaints in this cohort, we posit that there are myriad reasons why return visits could occur, including inadequate post-discharge communication regarding treatment plans, the inability of correctional health services to provide ED-recommended follow-up (such as medication changes, specialist referrals, or wound care) because of resource limitations, and the progression of incompletely treated conditions. In the general ED population, revisit rates have been widely studied, with a recent National Hospital Ambulatory Medical Care Survey (NHAMCS)-based analysis estimating 72-hour return rates of 4.5%-5.1% among admitted patients and a large cohort study of 9,036,483 patients reporting a 30-day revisit rate of 16.6% [19,20]. Although our cohort demonstrated a substantially lower 30-day revisit rate, this finding should be interpreted cautiously given the unique social, clinical, and system-level constraints faced by incarcerated patients.

Limitations 

This study has several limitations that should be considered when interpreting the findings. First, the use of payor source to identify incarcerated patients may not capture all ED encounters involving individuals who are incarcerated. Those with alternative insurance coverage (e.g., Medicaid or private insurance), those in federal custody whose care is billed through different payor channels, individuals incarcerated in local jails with different payor sources, or those whose correctional payor source was incorrectly recorded in the EHR may have been missed. Consequently, this method may be most effective for identifying individuals incarcerated in state correctional facilities whose care is billed through the state department of corrections. The true number of ED encounters involving incarcerated individuals during the study period may therefore be higher than reported. Second, this study was conducted at an academically affiliated health system in North Carolina, and the findings may not be generalizable to other geographic settings, nonacademic facilities, or states with different correctional health care funding and billing structures.

Third, the comparison cohort data were obtained using the Epic SlicerDicer tool, which provides aggregate population-level statistics rather than individual-level data. This limitation precluded formal inferential statistical testing (e.g., chi-square tests, t-tests, or regression analyses) of differences between the incarcerated and general adult ED cohorts. All comparisons presented are descriptive and should be interpreted accordingly. Future studies should use individual-level data for both cohorts to enable adjusted analyses that account for potential confounding variables.

Furthermore, the study period included the COVID-19 pandemic years (2020-2022), which significantly affected ED utilization patterns in both incarcerated and general populations. Pandemic-related changes in correctional health care delivery, visitation policies, and ED volume may have influenced the observed patterns. Finally, because the study relied on structured EHR data, we were unable to assess clinical outcomes, the quality of care delivered, or patient-reported experiences during ED encounters.

## Conclusions

This study demonstrates that payor source represents a feasible, low-cost, and replicable method for identifying ED encounters involving incarcerated individuals within EHR data, although further validation studies are needed before the method can be considered comprehensive for identifying incarcerated patients across health care systems. These findings contribute to a growing body of literature characterizing the ED care of people who are incarcerated and underscore the need for targeted interventions to address the unique health care needs of this medically underserved, socially marginalized, and vulnerable population.

Future research should validate the payor source identification approach across multiple health systems and states with varying correctional health care billing structures. Studies using individual-level comparison data should be conducted to enable formal statistical testing and multivariable analyses. Additionally, future work should examine clinical outcomes, quality metrics, and care processes for incarcerated patients in greater detail. The development and testing of targeted interventions, such as structured ED-to-correctional-facility discharge protocols, enhanced communication systems between health systems and correctional health services, and ED-based screening programs for hepatitis C and other prevalent conditions, could meaningfully improve continuity of care and health outcomes. By systematically identifying and characterizing this population, health systems can take important steps toward advancing health equity for people who are incarcerated.
